# Characterization of the Human Bitter Taste Receptor Response to Sesquiterpene Lactones from Edible Asteraceae Species and Suppression of Bitterness through pH Control

**DOI:** 10.1021/acsomega.0c05599

**Published:** 2021-02-01

**Authors:** Takuya Yanagisawa, Takumi Misaka

**Affiliations:** † Institute of Technology Solutions, R&D Division, Kewpie Corporation, 2-5-7, Sengawa-cho, Chofu-shi, Tokyo 182-0002, Japan; ‡ Department of Applied Biological Chemistry, Graduate School of Agricultural and Life Sciences, The University of Tokyo, 1-1-1 Yayoi, Bunkyo-ku, Tokyo 113-8657, Japan

## Abstract

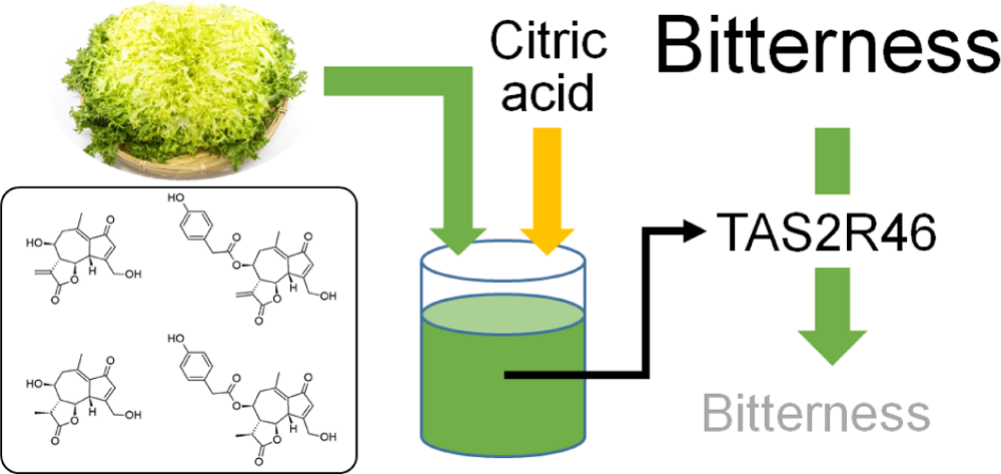

Vegetables are important sources of nutrients and bioactive compounds; however, their consumption is often insufficient, partly because of unpleasant taste characteristics. This study aimed to investigate the mechanisms underlying bitter taste reception and to develop methods to suppress bitterness. We focused on sesquiterpene lactones found in edible Asteraceae species. HEK293T cells that heterologously expressed human bitter taste receptors (including TAS2R46) together with a chimeric G protein were analyzed using calcium imaging, and cellular responses to four sesquiterpene lactones contained in lettuce were examined. We found that TAS2R46-expressing cells responded most strongly to bitter compounds. The EC_50_ value of 11β,13-dihydrolactucopicrin was 2.0 ± 0.6 μM, in agreement with the previously reported bitterness threshold of the compound. Adjustment of pH from neutral to weak acidic conditions reduced the response of TAS2R46-expressing cells to sesquiterpene lactones. We demonstrate the possibility of regulating the bitterness of Asteraceae species by controlling the pH.

## 1 Introduction

Vegetables are essential part of the human diet because they are sources of important micronutrients such as vitamins and minerals, as well as many other compounds that contribute to health. For example, polyphenols and terpenoids are ubiquitously present in plants and have been reported to have various bioactivities. Other examples of bioactive compounds of plant origin include anthocyanin, apiin, catechin, guaiazulene, momordicin, quercetin, and rutin, which have been reported to have health benefits through their antioxidant and anti-inflammatory properties,^1,2^ inhibition of carcinogenesis,^3,4^ and prevention of osteoporosis.^5^ Although increasing the consumption of vegetables is recommended, many people fall short of this recommendation, partly because of the unpleasant taste of some vegetables.

Many functional components of plant origin have strongly bitter tastes.^6,7^ For example, the bitterness threshold of cucurbitacin C—found in cucumbers—has been shown to be <0.1 mg/L,^8^ considerably lower than that of quinine hydrochloride,^9^ which is intensely bitter. Strong bitterness is generally perceived as an unpleasant taste; thus, altering the palatability of vegetables through the use of technology to control the bitterness may help to increase individuals’ consumption of vegetables. Recently, the relationship between preferences toward vegetables and sensitivity to bitterness has been explored, focusing on genotypical variations of the bitter taste receptor.^10^ Although the effects of strategies to mask bitterness on preferences for vegetables have been reported,^11^ the molecular mechanisms underlying the sensitivity to bitter compounds of plant origin are yet to be elucidated. A deeper understanding of these mechanisms would enable one to devise new methods to control the bitterness of vegetable. Tastants in ordinary foods are recognized by specific receptors in the oral cavity. Recently, molecular characterization of taste receptors has been conducted, and their functional features have been studied in detail.^12^ Bitter substances were detected by human type 2 taste receptors (TAS2Rs), which are G protein-coupled receptors present in taste bud cells of the tongue epithelium.^13−15^ Some TAS2Rs that bind to bitter components of plants, including vegetables, have been revealed,^16,17^ including salicin (which activates TAS2R16)^18^ and catechins (which activates TAS2R39);^19^ however, the receptors and associated mechanisms of detection have not been determined for many bitter compounds. Examples of such compounds are lactucin (Lac), lactucopicrin (LP), and 11β,13-dihydrolactucin (DHL), which are found in Asteraceae plants, such as chicory (*Cichorium intybus* L.)^9,20,21^ and lettuce (*Lactuca sativa* L.).^22^ These sesquiterpene lactones have sedative and analgesic effects,^23^ and the anti-malaria activity of Lac and LP has been confirmed.^24^ Although these compounds are strongly bitter,^22,25^ with bitterness thresholds equal to or higher than quinine hydrochloride,^9^ the mechanism underlying the detection of Lac, LP, and DHL has not yet been determined.

Controlling the bitterness of foods is important in the food industry, and methods to suppress bitterness have been developed on the basis of investigations into the underlying mechanisms of bitterness reception. Some compounds have been characterized, which act as inhibitors for the human bitter taste receptor; for example, 4-(2,2,3-trimethylcyclopentyl)butanoic acid (GIV3727) was identified by screening chemical compound libraries and has been shown to suppress the bitter aftertaste of artificial sweeteners, such as saccharin and acesulfame K.^26^ It has also been shown that the response of TAS2R16 to bitter disaccharides is suppressed by lowering the surrounding pH.^27^ In addition, it has been reported recently that some acidic amino acids and peptides can suppress the response of TAS2R16 to salicin.^28^

The purpose of this study was to investigate the mechanisms underlying bitter taste reception and to develop methods to control or suppress bitterness. We focused on bitter sesquiterpene lactones found in lettuce, for which the reception mechanisms are not clear. To this end, we investigated human bitter taste receptors known to respond to the bitter compounds in lettuce and evaluated the intensity of bitterness of the sesquiterpene lactones. We confirmed the suppression of bitterness using both cellular assays and human sensory tests.

## 2 Results and Discussion

### 2.1 Characterization of Human Bitter Taste Receptors that Respond to Sesquiterpene Lactones in Lettuce

To investigate the mechanisms underlying the reception of bitter taste of vegetables, we first examined the response of cells expressing human bitter taste receptors to LP, which is a well-known bitter sesquiterpene lactone found in lettuce. The presence of sesquiterpene lactones has been reported in Asteraceae species, and LP and Lac are known to be partly responsible for the bitterness of chicory^9^ and lettuce.^22^ From sensory tests, the bitterness threshold of LP has been reported to be 0.5 ppm (approximately 1.2 μM).^9^

**Figure 1:**
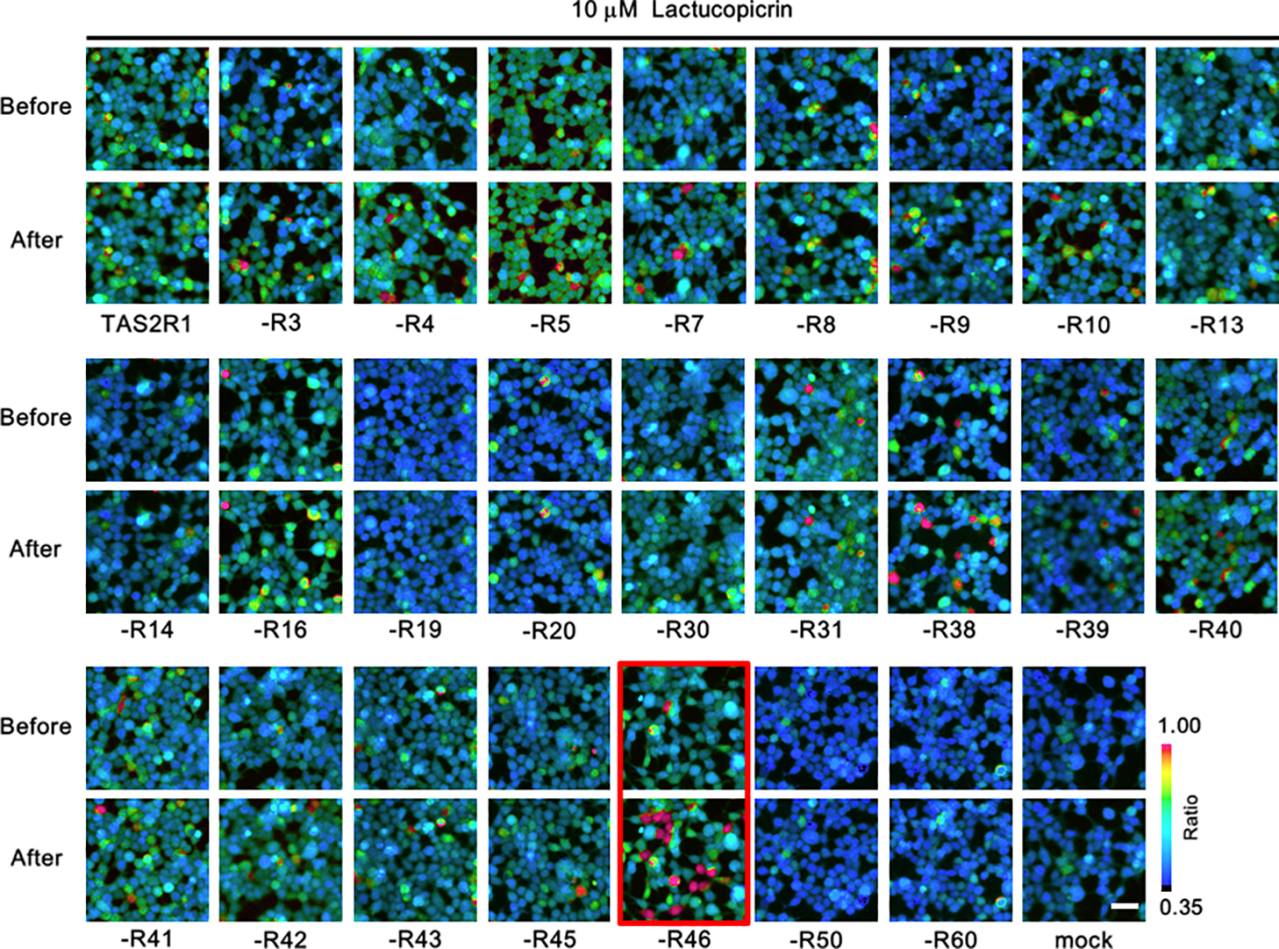
Representative ratiometric images of fura-2 loaded HEK293T cells expressing the human bitter taste receptors together with the chimeric G protein G16t2 following stimulation with 10 μM lactucopicrin. “Before” and “after” rows show representative cell images obtained before and after ligand application, respectively. “Rx” numbers below the images indicate the bitter taste receptor being investigated. The color scale indicates the F340/F380 ratio as a pseudocolor. Scale bar: 50 μm.

In the present study, we used a 10 μM LP solution, which should be sufficient considering the bitterness threshold of LP. Twenty-five kinds of HEK293T cells that express human bitter taste receptors and G16t2 were tested for cellular responses to 10 μM LP (Figure 1). We observed a clear cellular response from TAS2R46-expressing cells to LP (*p* < 0.0001, Tukey’s test), whereas cells expressing the other 24 species of human bitter taste receptor cells and mock cells showed no or only a faint response to 10 μM LP (Figure 1). These results strongly indicate that the bitterness of LP is primarily recognized by TAS2R46 in the oral cavity of humans because we confirmed a clear response at a concentration of about 10 times the bitterness threshold.

**Figure 2:**
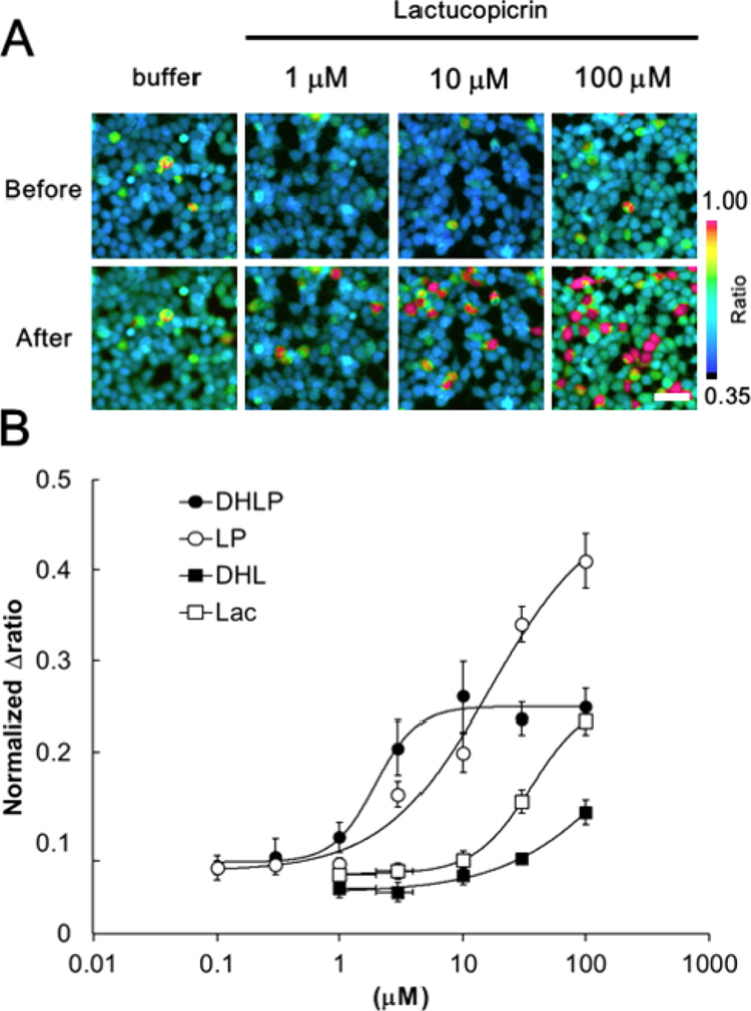
Responses of TAS2R46-expressing cells to four sesquiterpene lactones. (A) Representative ratiometric images of fura-2-loaded TAS2R46-expressing HEK293T cells to stimulation with 1, 10, and 100 μM lactucopicrin from Ca^2+^ imaging. “Before” and “after” rows show representative cell images obtained before and after ligand application, respectively. The color scale indicates the F340/F380 ratio as a pseudocolor. Scale bar: 50 μm. (B) Dose-dependent responses of TAS2R46-expressing cells to lactucopicrin (LP, open circles), DHLP (closed circles), lactucin (Lac, open squares), and DHL (closed squares). Data are presented as the mean; error bars show standard error of the mean from at least four independent experiments.

When LP was applied to cells expressing TAS2R46, weak but reproducible responses were detected after the addition of 1 μM LP (Figure 2A), indicating that the sensitivity of these cells is close to the bitterness threshold of humans. When the responses were examined quantitatively through calcium-imaging analysis, the responses were observed to be dose-dependent; the EC_50_ value of LP was calculated to be 16.6 μM (Figure 2B, LP).

### 2.2 Response of TAS2R46-Expressing Cells to Other Sesquiterpene Lactones Derived from Lettuce

**Figure 3:**
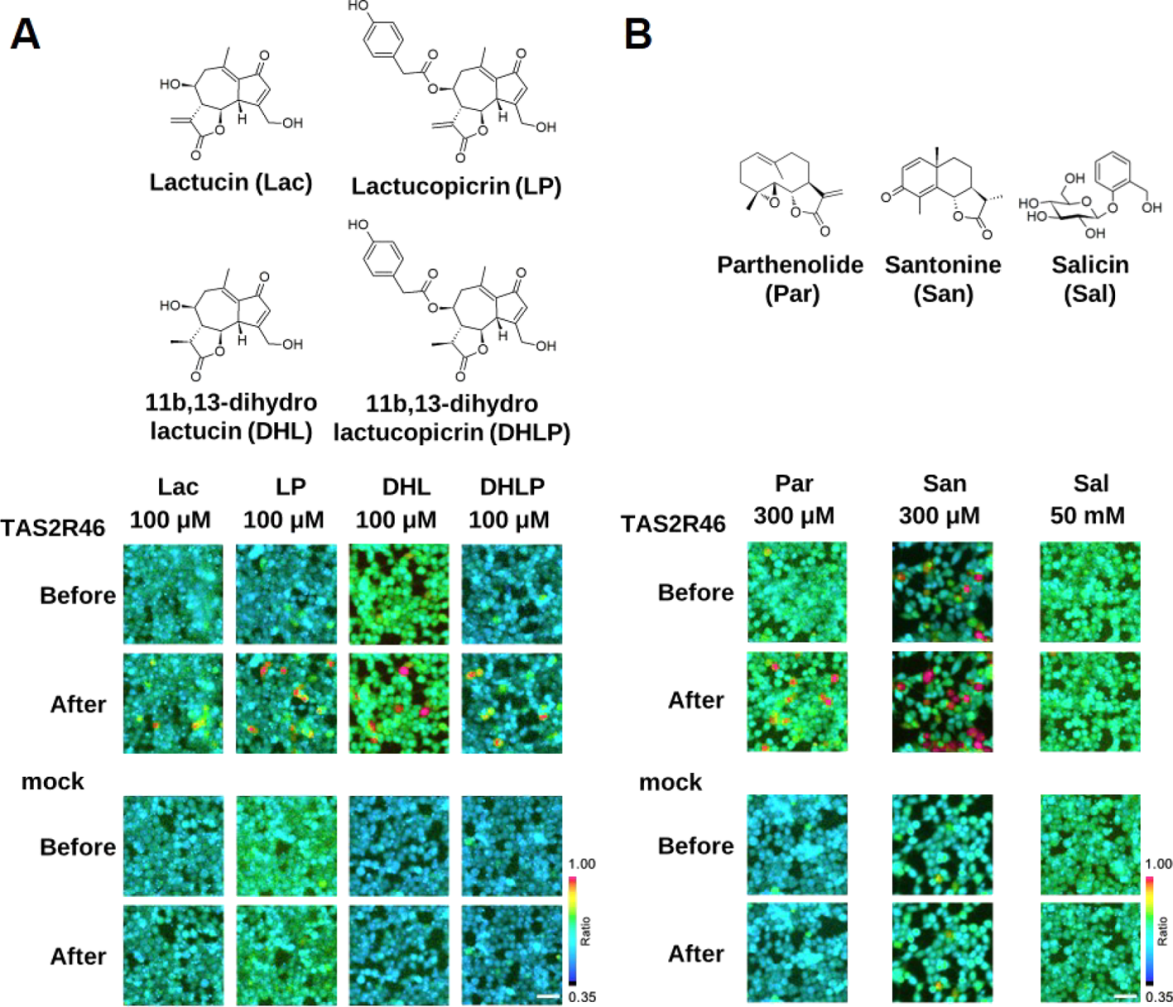
Representative ratiometric images of fura-2-loaded TAS2R46-expressing HEK293T cells and mock cells. To stimulation with (A) 100 μM Lac, LP, DHL, and DHLP and (B) parthenolide (300 μM), santonin (300 μM), and salicin (50 mM) from Ca^2+^ imaging. “Before” and “after” rows show representative cell images obtained before and after ligand application, respectively. The color scale indicates the F340/F380 ratio as a pseudocolor. Scale bar: 50 μm.

We also examined the application of other sesquiterpene lactones, which represent major bitter components of lettuce, to TAS2R46-expressing cells. It is reasonable to assume that other bitter compounds with a common chemical structure will induce a response in TAS2R46. We investigated the responses to DHLP, Lac, and DHL, whose chemical structures are similar in terms of the sesquiterpene lactone structure (Figure 3A). It should be noted that LP and DHLP also possess characteristic structures with the same functional group around the ester bond (Figure 3A).

Clear responses were detected when the four sesquiterpene lactones were examined at concentrations ranging from 0.1 to 100 μM, as in the case for LP (Figure 2B). Quantitative analysis showed that the responses were dose-dependent, with different effective concentrations determined for each compound (Figure 2B). Among them, DHLP induced a cellular response at the lowest concentration, with an EC_50_ of 2.0 ± 0.6 μM. Hence, responses to LP and DHLP agreed with the previously reported functional threshold,^9^ indicating the possibility that the bitterness intensity of the sesquiterpene lactones can be evaluated by the response profile of TAS2R46-expressing cells.

Moreover, we examined the response of TAS2R46-expressing cells to other compounds including parthenolide^29^ and santonin^30^ (TAS2R46 agonists) and salicin^18^ (TAS2R16 agonist) (Figure 3B). Parthenolide (300 μM) and santonin (300 μM) induced strong responses in TAS2R46-expressing cells, whereas no clear response was observed with salicin application (50 mM) (Figure 3B). This suggests that the sesquiterpene lactones including LP, DHLP, Lac, and DHL in lettuce appear to be selectively and specifically recognized by TAS2R46.

Compared with the results of LP and DHLP, the response of TAS2R46 to Lac and DHL was relatively low (Figure 2B). This might be attributed to the differences in the chemical structures; both LP and DHLP include 4-hydroxyphenyl acetic acid that is bonded to the hydroxyl groups of Lac and DHL, respectively (Figure 3A). The response of TAS2R46 clearly differs depending on the presence or absence of this structure (Figure 2B), suggesting its importance in ligand recognition by TAS2R46.

### 2.3 Suppression of the Bitterness Detection of Sesquiterpene Lactones by Controlling pH

We aimed to develop methods to control the bitterness of foods, such as vegetables. Previous reports proposed methods for the suppression of bitterness by inhibiting human bitter taste receptors. For example, the response of TAS2R16-expressing cells to gentiobiose (a rare, bitter-tasting disaccharide) was suppressed by the pH reduction of the ligand solution.^31^ Furthermore, the response of TAS2R16 to salicin was inhibited by adding acidic amino acids or umami peptides.^28^

**Figure 4:**
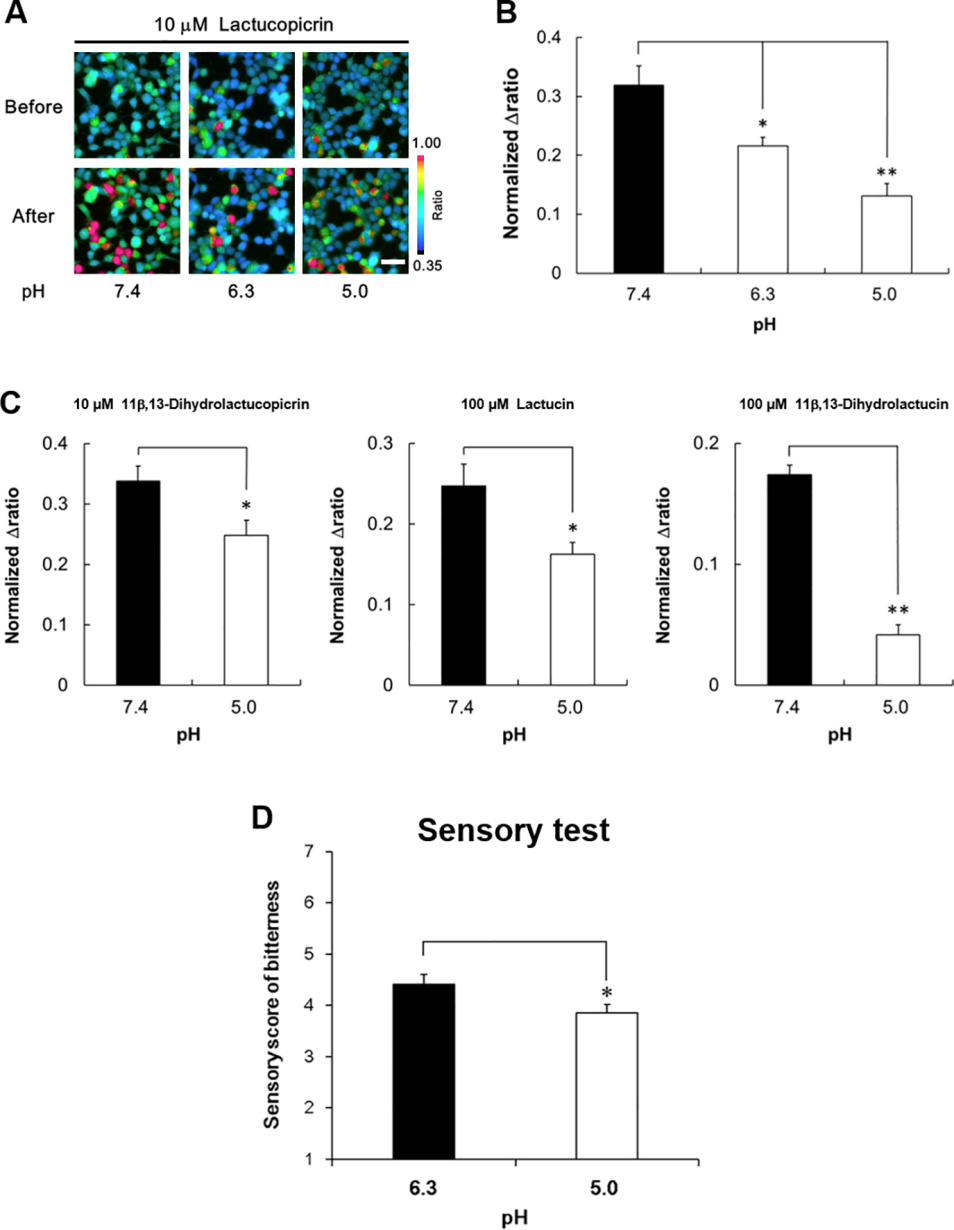
The suppression effects on bitterness of sesquiterpene lactones at low pH. (A) Responses of TAS2R46-expressing cells to 10 μM lactucopicrin under different pH conditions. “Before” and “after” rows show representative ratiometric images obtained before and after ligand application, respectively. The color scale indicates the F340/F380 fluorescence ratio as a pseudocolor. Scale bar: 50 μm. The cellular response in different pH conditions to (B) 10 μM lactucopicrin and (C) three sesquiterpene lactones. Data are presented as the mean; error bars show standard error of the mean from four independent experiments. The pH was adjusted with citric acid such that the pH after ligand application to the cells was equal to 6.3 or 5.0. The statistical significance of differences between the control (pH 7.4) and test results was determined using (B) one-way analysis of variance, followed by Dunnett’s test or (C) the Student’s *t*-test: *p < 0.05, **p < 0.01. (D) Sensory scores of bitterness for endive juice in different pH conditions. Sensory analysis was carried out using a trained panel with 27 members (13 males and 14 females). The two samples were evaluated using a paired comparison test, and the bitterness scored from 1 (very weak) to 7 (very strong) compared with the other sample (which was assigned 4 points). Data are presented as the mean; error bars show standard error of the mean. The statistical significance of differences between the scores of the two samples was determined using the Scheffé’s method of paired comparison using simple linear regression analysis: *p < 0.05.

In the present study, we examined the influence of pH conditions to establish whether this could reduce the response of TAS2R46 to sesquiterpene lactones. When the pH value of the LP solution was decreased by the addition of citric acid, the response of TAS2R46-expressing cells was attenuated compared with the control condition (pH 7.4) (Figure 4A). Quantitative analysis indicated that the cellular response at pH 6.3 and 5.0 was significantly decreased compared with that at pH 7.4. Because the cellular response to 10 μM LP at pH 5.0 (Figure 4B) was almost equivalent to that induced by 3 μM LP at pH 7.4 (Figure 2B), it can be considered that the bitterness of LP is greatly suppressed by a change in the pH from neutral to weakly acidic conditions.

Reductions in the effects of three other sesquiterpene lactones due to low pH were also examined (Figure 4C). The concentration of each sesquiterpene lactone was selected so as to induce a moderate cellular response from TAS2R46-expressing cells (Figure 2B). As a result, the cellular responses at pH 5.0 were significantly reduced compared with those at pH 7.4 at the same ligand concentrations in the case of all sesquiterpene lactones (Figure 4C), indicating that suppression of the bitterness of sesquiterpene lactones from lettuce can be achieved by controlling the pH.

In the experimental conditions of the present study, the inhibitory effects of decreased pH were in line with previous reports.^31^ In addition, the inhibitory effects under acidic conditions were confirmed for all four sesquiterpene lactones tested (Figure 4B,C), and no clear influence of chemical structure was observed. These results may indicate that suppressing TAS2R46 responses to sesquiterpene lactones is a feature shared between TAS2R46 and TAS2R16. On the other hand, it is also possible that this inhibitory effect occurs when taste information transmitted separately from taste receptors through taste nerves is integrated in the central nervous system. Therefore, it is necessary to carefully consider the possibility of peripheral and central effects on the suppression of bitterness due to sourness. Future experiments are required to determine whether the modulatory effect of pH is limited to select TAS2Rs.

### 2.4 Sensory Evaluation of Vegetable Juice in the Presence of Citric Acid

We investigated whether suppression of the response of TAS2R46-expressing cells to sesquiterpene lactones at low pH was maintained in human sensory evaluations. Endive (*Cichorium endivia*) juice was selected as a test sample due to its high content of sesquiterpene lactones. When the juice was adjusted to pH 5.0, the bitter taste was significantly reduced compared with the control juice (pH 6.3) (Figure 4D). This result suggests that the bitterness of lettuce can be suppressed by lowering the pH when eating through the use of acidic compounds. Notably, endive juice somewhat had a sour taste when the pH was lowered, but the bitter taste was perceived as significantly reduced compared with the control. In addition, further lowering of the pH resulted in members of the panel reporting increased sourness, and the evaluation of actual bitterness became difficult (data not shown).

The concentration of LP in endive juice was calculated from previously published data^32^ to be approximately 1.2–10 μM, which is equivalent to the concentration used in the cellular assay on TAS2R46-expressing cells in this study (Figures 1 and 2). The concentrations of DHLP, Lac, and DHL were estimated to be approximately 0.3–1.2, 2–3, and 7–12 μM, respectively. Considering the concentration–response curve for TAS2R46 (Figure 2B), the bitterness of endive juice perceived in sensory evaluations can be attributed to the presence of LP and DHLP. Although conditions in the oral cavity differ from those in our cellular experiments in terms of temperature, pH-buffering capacity of saliva, and so on, the suppression of responses of bitter taste receptors might also occur in the oral cavity.

Various studies have been conducted into the suppression of bitterness in vegetables and other foods, which have utilized physical methods, such as the removal of bitter components, suppression of bitter taste receptors with inhibitors,^26,33,34^ and masking of bitterness with other tastes.^11,35^ Because physical removal of the bitter components in vegetables also impairs the functional components, other methods, such as the adsorption of bitter components using polymer compounds^36^ or proteins,^37^ have been developed to prevent these from reaching the bitter taste receptors. In addition, masking bitterness with other strong tastes, such as sweetness, has been confirmed to have favorable effects.^38,39^ However, there is a certain degree of consumer resistance to the addition of excessive sweetness to vegetables.^11^

The results of this report, namely, that bitterness in vegetable juice can be effectively suppressed by lowering the pH, strongly indicate that the bitter taste of lettuce could be easily suppressed using sour seasonings such as vinegar, dressing, or mayonnaise in everyday meals. It is known that the newly targeted bitter taste receptor TAS2R46 responds to a wide range of plant-derived bitter components such as sesquiterpene lactones.^29^ In conclusion, the possibility of controlling food tastes may contribute to increasing vegetable intake among the general population without high costs.

## 3 Methods

### 3.1 Chemicals

DHL, 11β,13-dihydrolactucopicrin (DHLP), Lac, and LP were purchased from Funakoshi Co., Ltd. (Tokyo, Japan). Santonin and parthenolide were obtained from Tokyo Chemical Industry Co. Ltd. (Tokyo, Japan). Compounds were dissolved in a mixture of dimethyl sulfoxide (DMSO) and assay buffer to a final DMSO concentration of ≤0.1% (v/v) to avoid toxic effects on cultured cells.

### 3.2 Reagents

Ligands were diluted into assay buffer [10 mM 4-[2-hydroxyethyl]-1-piperazineethanesulfonic acid, 130 mM NaCl, 10 mM glucose, 5 mM KCl, 2 mM CaCl_2_, and 1.2 mM MgCl_2_ (pH adjusted to 7.4 using NaOH)] at the desired concentrations.

### 3.3 Cell Culture and Transfection

The construction of expression plasmids for TAS2Rs has been reported previously.^27^ Briefly, the nucleotide sequence for the first 45 amino acids of rat somatostatin receptor type 3 (sstr3) was ligated to the 5′-terminus of the coding region of the gene for each TAS2R. A chimeric G protein (G16t2) was designed, which replaced the 44 amino acids at the C terminus of human Gα16 with those from zebrafish Gαt2 (refseq: NM_131869). The resultant cDNA was subcloned into a pEAK10 expression vector (Edge Biosystems, Gaithersburg, MD, USA).

HEK293T cells were cultured at 37 °C in Dulbecco’s modified Eagle’s medium (Sigma-Aldrich Japan, Tokyo, Japan) supplemented with 10% fetal bovine serum (Thermo Fisher Scientific, Waltham, MA, USA). Cells were seeded into six-well plates and then transiently transfected with the plasmids expressing sstr3-TAS2Rs, G16t2, and red fluorescent protein (pDsRed2-N1; Takara Bio, Shiga, Japan) in the ratio of 40:10:0.8 using Lipofectamine 2000 (Thermo Fisher Scientific).

### 3.4 Ca^2+^ Imaging

Six hours after transfection, the cells were seeded into 96-well plates (Lumox multiwell 96-well, SARSTEDT AG and Co., Nümbrecht, Germany), after which they were incubated for an additional 18–20 h. Subsequently, cells were washed with assay buffer; then, 5 μM of fura-2-acetoxymethyl ester (fura-2 AM; Thermo Fisher Scientific) was added into the medium and incubated for 30 min at 27 °C. The cells were then washed with assay buffer and incubated for an additional 15 min at room temperature.

Ligands were manually administered into the well by adding 100 μL of aliquots of assay buffer supplemented with 2× ligands at the desired concentrations. Fura-2 fluorescence intensities were measured by excitation at 340 and 380 nm, followed by detection at 510 nm using a Lambda 10-3 computer-controlled filter changer (Sutter Instruments, San Rafael, CA, USA), a CoolSNAP HQ2 camera (Photometrics, Tucson, AZ, USA), and an IX-81 inverted fluorescence microscope (Olympus, Tokyo, Japan). Images were recorded at 4-s intervals and analyzed using the MetaFluor software (Molecular Devices, Sunnyvale, CA, USA). Changes in intracellular calcium ion concentrations were measured by randomly selecting DsRed2-positive cells, which were considered to be transfected cells. Changes in fluorescence are presented as the ratio of the fluorescence intensities at the two excitation wavelengths (F340/F380). The fluorescence intensity ratio at the start of imaging of each cell was defined as “ratio (at time = 0)”. The difference between the maximum fluorescence intensity ratio within 48 s of imaging and “ratio (at time = 0)” was defined as “Δratio”. “Normalized Δratio” was defined as the average value of Δratio/[ratio (at time = 0)] for 100 cells in one field. For each measurement condition, the average normalized Δratio for four to six independent wells was calculated. Half-maximal effective concentration (EC_50_) values were calculated from dose–response data using Clampfit 9.2 (Molecular Devices) using Hill’s equation.

When the pH was changed to create acidic conditions, the pH levels of the ligand solutions were adjusted with citric acid such that the pH of the medium after ligand application to the cells was equal to 6.3 or 5.0.

### 3.5 Sensory Analyses

Sensory tests using conventional foods were approved by the Research Ethics Committee, R&D Div., Kewpie Co. (2013), and the panel provided informed consent for participation.

Endive (*Cichorium endivia*) samples were used for sensory analysis because this plant is the most bitter of the commercially available Asteraceae vegetables that contain sesquiterpene lactones. A total of 600 g of endive was added to 800 mL of water and milled with a mixer (SM-L56, Panasonic Co., Osaka, Japan). After filtration with a 20-mesh polyethylene terephthalate filter cloth, 1.2 kg of endive juice was obtained. The juice was divided equally into two portions; one was adjusted to pH 5.0 with citric acid (A), whereas the other was not adjusted for pH (B; its pH value was measured to be 6.3).

A trained panel of 27 members (13 males and 14 females) was used for taste tests. These members were selected by a concentration difference discrimination test using caffeine. The test implementation was performed only in the panel that provided consent. The two samples were evaluated using a paired comparison test; then, the bitterness of each sample was scored from 1 (very weak) to 7 (very strong) compared with the other sample as a control, which was awarded a score of 4. Fourteen panel members evaluated the bitter taste of sample (A) compared with sample (B) as the control sample, whereas the remaining 13 evaluated the bitterness of sample (B) compared with sample (A) as the control sample.

### 3.6 Statistical Analysis

Statistically significant differences were determined using Student’s *t*-test, one-way analysis of variance, followed by Dunnett’s test, Tukey’s test, or Scheffé’s method of paired comparison.^40^ We considered *p*-values of <0.05 to indicate statistically significant differences. JMP 14.0 (SAS Institute, Cary, NC) was used for statistical analyses.

## Abbreviations

DHL: 11β,13-dihydrolactucin
DHLP: 11β,13-dihydrolactucopicrin
DMSO: dimethyl sulfoxide
Lac: lactucin
LP: lactucopicrin
